# A randomised clinical trial of methotrexate points to possible efficacy and adaptive immune dysfunction in psychosis

**DOI:** 10.1038/s41398-020-01095-8

**Published:** 2020-11-30

**Authors:** I. B. Chaudhry, M. O. Husain, A. B. Khoso, M. I. Husain, M. H. Buch, T. Kiran, B. Fu, P. Bassett, I. Qurashi, R. ur Rahman, S. Baig, A. Kazmi, F. Corsi-Zuelli, P. M. Haddad, B. Deakin, N. Husain

**Affiliations:** 1 Faculty of Biology, Medicine and Health, Division of Neuroscience and Experimental Psychology, School of Biological Sciences, University of Manchester, Manchester Academic Health Science Centre, Manchester, M13 9PT UK; 2 Dow University of Health Sciences, Karachi, Pakistan; 3 Ziauddin University Hospital, Karachi, Pakistan; 4 Centre for Addiction and Mental Health, Toronto, ON Canada; 5 Department of Psychiatry, University of Toronto, Toronto, ON Canada; 6 Pakistan Institute of Living and Learning, Karachi, Pakistan; 7 National Institute of Health Research Leeds Biomedical Research Centre, Leeds Teaching Hospitals NHS Trust, Leeds, UK; 8 Institute of Cellular Medicine, Newcastle University and National Institute for Health Research Newcastle Biomedical Research Centre, Newcastle upon Tyne Hospitals NHS Foundation, Newcastle upon Tyne, UK; 9 School of Data Science, Fundan University, Shanghai, China; 10 Stats Consultancy, Amersham, UK; 11 Faculty of Biology, Medicine and Health, Division of Psychology and Mental Health, School of Biological Sciences, University of Manchester, Manchester Academic Health Science Centre, Manchester, M13 9PT UK; 12 Baqai University, Karachi, Pakistan; 13 Karwan e Hayat, Karachi, Pakistan; 14 Division of Psychiatry, Department of Neuroscience and Behaviour, Ribeirão Preto Medical School, University of São Paulo (FMRP—USP), São Paulo, São Paulo 14048-900 Brazil; 15 Hamad Medical Corporation, Doha, Qatar

**Keywords:** Schizophrenia, Neuroscience

## Abstract

NMDA autoantibody encephalitis presenting as schizophrenia suggests the possible role of adaptive cell-mediated immunity in idiopathic schizophrenia. However, to our knowledge there have been no trials of the immune-suppressant methotrexate in schizophrenia. We tested if low-dose methotrexate as used in the treatment of systemic autoimmune disorders would be tolerable and effective in people with schizophrenia in a feasibility study. Ninety-two participants within 5 years of schizophrenia diagnosis were recruited from inpatient and outpatient facilities in Karachi, Pakistan. They were randomised to receive once weekly 10-mg oral methotrexate (*n* = 45) or matching placebo (*n* = 47) both with daily 5-mg folic acid, in addition to treatment as usual for 12 weeks. There were eight dropouts per group. Side effects were non-significantly more common in those on methotrexate and were not severe. One person developed leukopenia. Positive symptom scores improved more in those receiving methotrexate than placebo (*β* = −2.5; [95% CI −4.7 to −0.4]), whereas negative symptoms were unaffected by treatment (*β* = −0.39; [95% CI −2.01 to 1.23]). There were no immune biomarkers but methotrexate did not affect group mean leucocyte counts or C-reactive protein. We conclude that further studies are feasible but should be focussed on subgroups identified by advances in neuroimmune profiling. Methotrexate is thought to work in autoimmune disorders by resetting systemic regulatory T-cell control of immune signalling; we show that a similar action in the CNS would account for otherwise puzzling features of the immuno-pathogenesis of schizophrenia.

## Introduction

A number of findings suggest that inflammatory processes are involved in the pathogenesis of schizophrenia. Many studies report raised circulating cytokine and C-reactive protein (CRP) concentrations in patients with schizophrenia^1^. Meta-analyses confirm that acutely ill patients with schizophrenia have raised plasma concentrations of CRP and classic inflammatory cytokines such as IL-6 and TNF-α in comparison with non-psychotic samples^2^. These findings have led to much interest in the possibility that peripheral changes might induce or reflect inflammatory changes in the brain that could be reversed by anti-inflammatory drugs. Other studies suggest that autoimmune mechanisms may be at play. Epidemiological studies report that patients with schizophrenia and their relatives have an increased risk of autoimmune disorders such as systemic lupus erythematosus and psoriasis^3^. More directly, encephalitis with autoantibodies against the NMDA glutamate receptor can present as schizophrenia^4^, however, such antibodies are not common in sporadic schizophrenia^5^. Nevertheless, genetic loci in the major histocompatibility complex are clearly associated with psychosis risk, and genes in this region regulate cell-mediated immune mechanisms of the adaptive immune system and mechanisms of autoimmunity^6^.

These lines of evidence have led researchers to investigate the adjunctive use of anti-inflammatory agents in the treatment of schizophrenia. In a meta-analysis, two studies of aspirin in 136 patients found that positive symptoms improved with the addition of aspirin compared to antipsychotic treatment alone^7^. However, the selective COX-2 inhibitor celecoxib showed no overall benefit on symptoms. The antibiotic minocycline has known anti-inflammatory effect in autoimmune disorders such as systemic lupus erythematosus and rheumatoid arthritis, although it is used infrequently today. Furthermore, minocycline is known to reduce the inflamed state of peripheral macrophages and their brain-resident equivalents, microglia^8^. A number of small clinical trials have reported that minocycline improves negative symptoms when added to treatment as usual (TAU) in schizophrenia, including our proof of concept 2-centre study in Pakistan and Brazil^8^. However, in a definitive follow-up UK study in 207 patients with recent onset psychosis, we found no benefit of minocycline after 8 or 52 weeks of treatment^9^. The reasons for the discrepant findings are not clear but another large study in 200 patients with established illness also reported no benefit of minocycline^10^. In the UK study, plasma cytokine levels did not predict outcome and did not change over the course of treatment. Magnetic resonance imaging (MRI) revealed no decreases in grey matter to suggest neuropathic changes over 12 months in the placebo group. There was thus little evidence, albeit indirect, of an active neuroinflammatory process in the patient sample. More directly, a number of positron emission tomography (PET) studies in patients at various stages of schizophrenia now report no increase in radioligand binding to the translocator protein (TSPO), which is a biomarker for activated microglia^11^. Our recent study found that reductions in TSPO binding in schizophrenia, which we hypothesised, are an indication that microglia are in a non-inflamed phagocytic mode driven by astrogliosis^12^. These findings together with large scale transcriptomic evidence in post-mortem brain, discussed below, suggest that microglial inflammation may not be central to pathogenesis in schizophrenia, hence the lack of efficacy of minocycline.

We carried out a trial to test the feasibility of evaluating the efficacy of methotrexate, a potent immune-suppressant drug that acts on cell-mediated adaptive immunity with indirect anti-inflammatory actions on the innate immune system. At high doses (up to a gram/dose) the anti-folate actions of methotrexate mediate its anti-proliferative effects through inhibition of purine and pyrimidine synthesis. However this mechanism is minimised by the use of low doses (7.5–25 mg/week) combined with folate supplements in routine therapy for autoimmune disorders such as rheumatoid arthritis and psoriasis^13^. These disorders are associated with impaired control of the immune response by circulating regulatory T cells (Tregs) and thus a pro-inflammatory balance in functional T-cell populations and in the pattern of cytokine secretion^14,15^. Methotrexate restores impaired Treg function in-vitro and during treatment for example, through resetting epigenetic control of FoxP3 expression, the master regulator of Treg function^16^. Methotrexate is known to increase tissue levels of adenosine, a potent anti-inflammatory molecule, and this is also mediated by Tregs^14^. These actions restore an anti-inflammatory cytokine profile in autoimmune diseases^3,17^. Given the well-known comorbidity and shared familial risk between schizophrenia and autoimmune disorders^18,19^, the possible antipsychotic efficacy of methotrexate is of considerable aetiological significance for schizophrenia. We report the first trial evaluating the feasibility and efficacy of methotrexate added to TAU in patients with early schizophrenia spectrum disorders within 5 years of onset—when neuroinflammation might still be active with less exposure to antipsychotic drugs. The results have been published in abstract form and we are not aware of other trials of methotrexate in patients with psychotic illness^20^.

## Methods

### Study design and participants

We conducted a randomised, double blind, placebo-controlled exploratory trial of methotrexate 10 mg once a week added to TAU for patients with schizophrenia, schizoaffective disorder, psychosis not otherwise specified and schizophreniform disorder. The trial was registered on Clinicaltrial.gov (NCT02074319) on February 28, 2014. The final protocol and the feasibility and statistical analysis plan were published in 2015^21^.

The study was completed between December 2013 and August 2015 at four major hospitals in Karachi, Pakistan. The Pakistan Institute of Living and Learning independent Ethics Committee approved the study (Project Reference: PILL/SMRI/12627). The study was conducted in accordance with the principles of the Declaration of Helsinki. All patients gave their written, informed consent prior to enrolment in the study.

Participants were recruited from inpatient and outpatient psychiatric departments. Participants between the ages of 18 and 35 years, meeting the Diagnostic and Statistical Manual-IV (DSM-IV) criteria for schizophrenia, schizoaffective disorder, psychosis not otherwise specified or schizophreniform disorder and within the first 5 years of diagnosis were eligible to take part in the study. All participants included in the trial were on stable medication for 4 weeks prior to baseline assessments. Female participants were included in the study if they agreed to continue adequate contraception during the trial.

Exclusion criteria included: organic brain disease or a neurological diagnosis; significant renal or hepatic impairment; pre-existing blood dyscrasias (marrow hypoplasia, leukopenia, thrombocytopenia or anaemia); DSM-IV criteria for substance misuse in the previous 6 months (other than nicotine) or use of psychotropic drugs of abuse in the prior month (other than nicotine); pregnant or lactating women and those of reproductive age but not using adequate contraception.

Treating clinical teams initially identified and approached potential participants meeting entry criteria for the trial, provided a patient information leaflet about the study and offered to arrange a visit from the research team to explain the study in more detail. Potential participants were given at least 24 h after the detailed explanation by a member of the research team before they made a decision on whether to give signed consent to participate in the study and to provide consent for access to participant medical records.

### Randomisation and masking

Participants were allocated to active methotrexate or matching placebo according to a randomised permuted blocks algorithm with stratification by treating hospital site. Patients were allocated to either methotrexate or placebo according to pseudo-random sequence generated by the trial statistician in Manchester to produce roughly equal groups. The details of the allocation were concealed from the research team until all data collection had been completed. The study pharmacist dispensed the medication on a fortnightly basis. In the event of a medical emergency, only the trial pharmacist or deputy had access to the treatment allocation list for unblinding and this would only proceed if the chief investigator or his deputy authorised it.

Patients took trial medication of methotrexate 10 mg orally once a week or matching placebo for the duration of the trial (12 weeks). All participants in both arms of the trial also took folic acid 5 mg/day orally for 6 days a week except the day that trial medication (methotrexate or placebo) was given. No restrictions were placed on medication changes (type or dose), though stability in medication regime was encouraged. TAU consisted of first- or second-generation antipsychotic medication, as deemed suitable by their responsible psychiatrist. There was minimal access to formal psychological therapies. The consultant psychiatrist for each participant remained responsible for his or her clinical care.

### Procedures

The schedule of assessments is detailed in the published protocol^21^. At screening, the trained research assistants (RAs) confirmed diagnostic criteria on the basis of the Structured Clinical Interview for DSM-IV. Urine was obtained for pregnancy tests. At the randomisation visit, all baseline symptomatic, cognitive and functional efficacy measures were recorded, together with assessments of side effects and medication adherence. Symptomatic measures, side effects and adherence assessments were repeated at follow-up visits at 2, 4, 8 and 12 weeks. Even low-dose methotrexate can have serious side effects^22^ and we monitored signs of toxicity with a checklist based on UK guidelines^23^. We checked haematological measures, renal function, liver function and routine CRP at baseline, 2, 4, 6 and 12 weeks. At 12 weeks, the baseline cognitive tasks and measures of functioning and quality of life were repeated. Project RAs had regular training and harmonisation discussions at 2-weekly teleconferences and at 6 monthly away days.

The trial was monitored by an independent Trial Steering Committee (TSC) that included a senior physician and a service user. The TSC also had the responsibility for data monitoring to oversee any potential harm to the participants from taking part in the trial.

### Outcomes

The feasibility outcomes were recruitment and retention rates, a checklist of common side effects experienced with methotrexate, and adherence (pill check) to the trial medication. We measured efficacy using the positive and negative symptom subscale scores and the total score on the Positive and Negative Syndrome Scale (PANSS)^24^. Efficacy outcomes also included assessments of functioning: Global Assessment of Functioning (GAF)^25^; Schedule of Assessment for Insight (SAI)^26^; Clinical Global Impression Scale (CGI)^27^; EuroQol-5D (EQ-5D)^28^ and the Social Functioning Scale (SFS)^29^. Cognition was assessed using “pencil and paper tests” and CogState^30^. The assessments included processing speed, attention/vigilance, working memory (nonverbal and verbal), verbal learning, visual learning, reasoning and problem solving and social cognitions. These assessments covered all seven domains recommended by MATRICS (NIMH initiative)^31^. Adverse effects were monitored using a checklist specifically designed for methotrexate.

### Statistical analysis

There are no studies available concerning the effect of methotrexate added to TAU in early schizophrenia to inform an optimal sample size. The primary aim of this study was to determine acceptability and tolerability of methotrexate added to TAU but we also aimed to provide efficacy data to better estimate the sample size for future trials. Our sample size consideration was based on the hypothesis that there is a significant difference on the clinical outcome measures (e.g., PANSS) and the cognitive function measures from baseline to end point between the methotrexate group and TAU group. The sample size was calculated to detect a group difference at *p* < 0.20 due to the exploratory nature of the study. With 32 participants per group, this study would have 80% power to detect a medium standardised effect size of 0.53. The estimated loss to follow-up rate was 10% and, therefore, a total of 72 patients (36 methotrexate, 36 TAU) were needed.

Differences between treatment groups for the final 12-week clinical outcome measures and cognitive function measures were analysed using analysis of covariance, with baseline measures as covariates, as described in the published protocol^21^. Outcomes with strongly positively skewed distributions were log-transformed. In the secondary planned analysis, we analysed the repeated clinical outcome measures at baseline, 2, 4, 8 and 12 weeks using generalised estimating equations (GEE) to estimate the effect of treatment on longitudinal outcomes, including baseline values and time terms as covariates taking account of the within-subject correlation in longitudinal data using SAS 9.4 (SAS Institute Inc., Cary, North Carolina, USA). Multiple imputation was used to address the influence of missing values on outcome using all data from all 92 randomised participants in an intention to treat sensitivity analysis based on the multivariate normal distribution method^32^. Thirty imputed samples were created per missing final value and analysed simultaneously using Stata version 15.1. The occurrence of side effects was compared between the two treatment groups by Chi square.

## Results

### Recruitment and retention

**Fig. 1:**
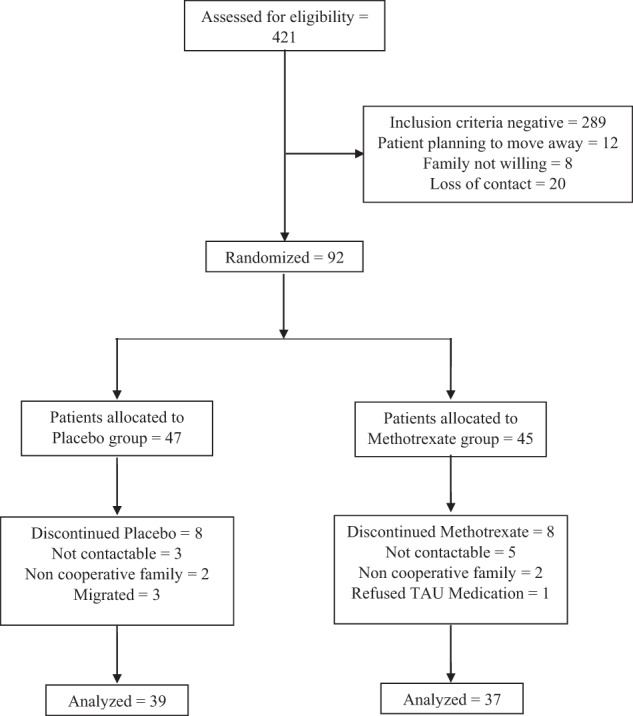
Patient flow through the study. Consort diagram.

We approached 421 participants from December 2013. The date of first randomisation was January 2014 and the last visit was completed in June 2015. These participants were then assessed for eligibility to take part in the trial (Fig. 1). Of these, 289 were excluded as they did not fulfil the inclusion criteria, 8 were not willing to take part, 12 were planning on moving away from the city and 20 were not contactable following the initial screening visit. This left 92 participants who were randomly allocated to either receive methotrexate (*n* = 45) or placebo (*n* = 47), in addition to TAU. Recruitment continued for longer than initially planned, as dropouts were higher than expected. Seventy-six participants completed the study, 39 in the placebo arm and 37 in the methotrexate arm, slightly overshooting the 32 per arm target. The retention rate in both arms was ~80%. There were an equal number of dropouts in both groups (*n* = 8). Following randomisation eight participants discontinued the TAU arm, with three participants uncontactable, three migrating and two families refusing to continue with the trial. In the methotrexate arm, five participants were uncontactable, two families refused to continue with the study, and one participant refused their TAU medication.

### Baseline characteristics

The baseline characteristics were similar in the intervention and control groups (Table 1). The mean (SD) age of participants was 24.8 (4.2) years in the treatment arm and 26.6 (4.9) years in the control group. All were outpatients. There was a male predominance in both groups with ~78% males in the intervention arm and 68% in the placebo. Educational attainment in both arms was limited to 6 or 7 years in both groups. The majority (72%) of participants were single. Diagnosis in over half of both groups, according to DSM-IV criteria, was paranoid schizophrenia. Twelve people had disorganised, residual or schizoaffective subtypes and most (*n* = 10) were allocated to placebo, they were eliminated in a secondary analysis below. The mean total PANSS was in the mild–moderate range for both groups; methotrexate group 65 and placebo 59. CGI severity scores at baseline also did not show any significant group difference. All but five patients were on risperidone or olanzapine and treatments were evenly distributed between the groups (Table ST1).

**Table 1:** Characteristics of the sample.

	Methotrexate (*n* = 45)	Placebo (*n* = 47)
	Mean (SD)	Mean (SD)
Age (years)	24.8 (4.2)	26.6 (4.9)
Education (years)	5.7 (4.0)	7.0 (4.0)
Clinical Global Impression (CGI)	4.5 (0.8)	4.4 (0.8)
PANSS (subscale scores)		
Positive symptoms	18.0 (5.9)	15.7 (6.3)
Negative symptoms	16.4 (4.4)	15.0 (3.7)
General psychopathology	30.4 (7.8)	28.4 (4.9)
Total score	64.8 (15.1)	59.0 (11.4)

	*N* (%)	*N* (%)
Sex		
Male	35 (77.8)	32 (68.1)
Female	10 (22.2)	15 (31.9)
Marital Status		
Single	29 (64.4)	37 (78.7)
Married	15 (33.3)	9 (19.2)
Separated	0 (0.0)	1 (2.1)
Divorced	1 (2.2)	0 (0.0)
Diagnosis of SCID		
Paranoid type	32 (71.1)	28 (59.6)
Disorganised type	2 (4.4)	6 (12.8)
Undifferentiated type	11 (24.4)	9 (19.1)
Residual type		3 (6.4)
Schizoaffective disorder		1 (2.1)

### Clinical and cognitive outcomes

**Fig. 2:**
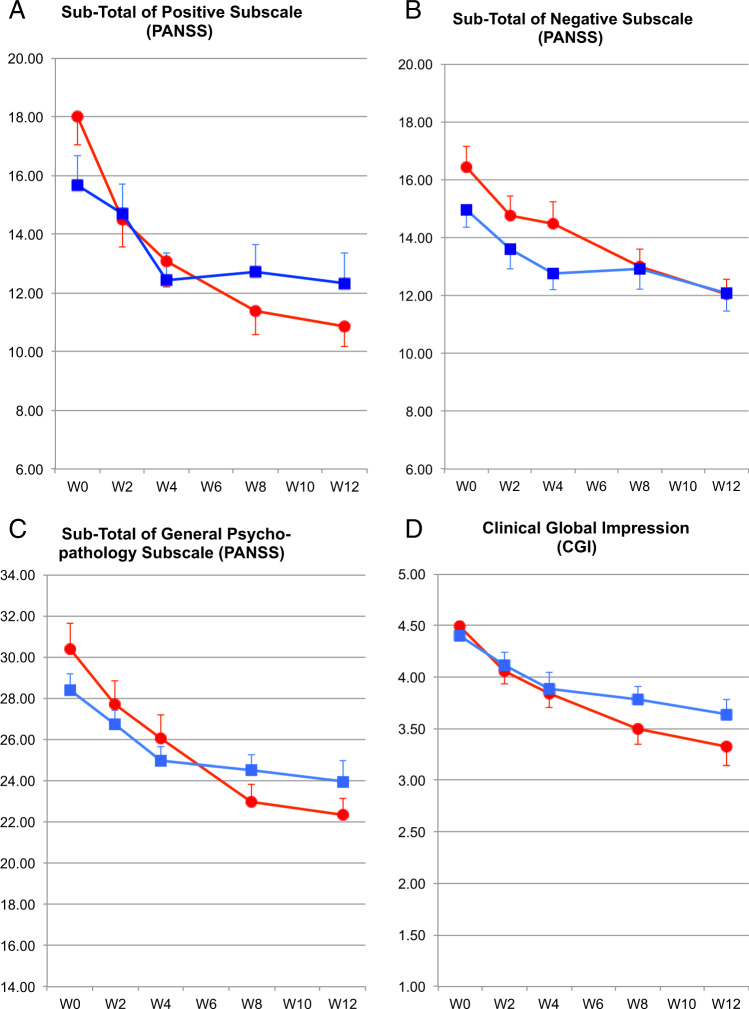
Mean clinical outcome trajectories by randomisation group.

Both groups showed an improvement on all the major clinical outcomes during the study (Fig. 2). The total PANSS scale score dropped by 30% in the methotrexate group and 18% in the placebo group. Methotrexate had a statistically significant effect on PANSS Positive Subscale (*p* = 0.02) and GAF Scale (*p* = 0.03). There was a trend indicative of improvement in PANSS General Psychopathology Subscale (*p* = 0.06) and PANSS total score was of borderline significance (*p* = 0.05). There was no statistically significant effect on PANSS negative subscale, CGI, EQ-5D, SAI or SFS. However, on the six social domains of the SFS, methotrexate-associated improvement was seen in pro-social activities (*p* = 0.03), recreational activities (*p* = 0.06) and interpersonal behaviour (*p* = 0.08) but with no numeric benefit on performance of living skills or on social engagement (Table ST2). The treatment effects are summarised in Table 2. There were no statistically significant effects of treatment on the cognitive parameters (Table 3). There was no statistically significant change in white cell count or CRP between the two groups (Table 2). The results were confirmed by the GEE analysis using all the intermediate PANSS scores, with a slightly greater treatment effect of −2.94 (95% CI −5.10, −0.77) and significance level (*p* = 0.008) for the PANSS positive scores and no significant change in PANSS negative scores −0.74 (−2.36, 0.87), *p* = 0.366. The group difference remained statistically significant in the ITT sensitivity analysis (−2.2 (95% CI: −4.5 to 0.0); *p* = 0.049). We repeated the efficacy analysis excluding the 12 participants with disorganised, residual or schizoaffective subtypes; the efficacy of methotrexate remained unchanged (−2.52 (95% CI: −4.9 to 0.09); *p* = 0.042).

**Table 2:** Clinical outcomes: ratings and treatment effects at end point.

	MTX			Placebo			Treatment effect	
	*N*	Baseline Mean (SD)	3 months Mean (SD)	*N*	Baseline Mean (SD)	3 months Mean (SD)	Mean (95% CI)	*P*
Positive symptoms	37	18.0 (5.9)	10.9 (4.2)	39	15.3 (6.4)	12.3 (6.3)	−2.5 (−4.7, −0.4)	0.02
Negative symptoms	37	16.5 (4.6)	12.0 (3.4)	39	14.7 (3.7)	12.1 (3.8)	−0.4 (−2.0, 1.3)	0.64
General psychopathology	37	30.0 (7.9)	22.3 (4.9)	39	27.8 (4.2)	24.0 (6.0)	−2.3 (−4.7, 0.1)	0.06
PANSS total score	37	64.0 (15.5)	45.2 (10.2)	39	57.8 (10.4)	48.4 (13.6)	−5.3 (−10.6, −0.1)	0.05
Clinical Global Impression	37	4.4 (0.8)	3.3 (0.9)	39	4.3 (0.70)	3.6 (1.1)	−0.3 (−0.8, 0.1)	0.13
Global Assessment of Functioning Scale	37	5.6 (1.0)	4.2 (1.0)	39	5.3 (1.1)	4.5 (1.2)	−0.5 (−1.0, 0.0)	0.03
Social Functioning Scale	37	82.6 (25.3)	103.7 (26.6)	39	85.6 (27.1)	95.7 (26.9)	8.8 (−2.4, 20.0)	0.11
SAI: total score	37	8.0 (5.0)	11.9 (4.3)	39	7.2 (5.2)	10.6 (4.2)	1.0 (−0.8, 2.8)	0.26
EQ-5D: VAS	37	58.1 (28.4)	65.5 (24.8)	39	61.8 (27.5)	67.6 (23.0)	−1.6 (−12.5, 9.4)	0.78
C-reactive protein^a^	36	6 [5,6]	6 [6, 6]	36	6 [6, 6]	6 [6, 6]	1.02 [0.97, 1.07]	0.40
White cell count (/ml)	37	6911 (1739)	6802 (1702)^b^	39	7033 (2169)	7227 (1845)^b^	−419 (−1170, 333)	0.13

*PANSS* Positive and Negative Syndrome Scale, *SAI* Schedule of Assessment for Insight, *EQ-5D VAS* EuroQol-5D Visual Analogue Scale.

^a^Due to a skewed distribution, median [inter-quartile range] reported at each timepoint, along with ratio of values between groups [95% CI].

^b^Two in each group refused final blood sample.

**Table 3:** Cognitive function: treatment effects at end point.

	MTX			Placebo			Treatment effect	
	*N*	Baseline Mean (SD)	3 months Mean (SD)	*N*	Baseline Mean (SD)	3 Months Mean (SD)	Mean (95% CI)	*P*
Stroop Test Word (s)	24	141 (65)	112 (50)	27	120 (62)	101 (43)	1 (−20, 22)	0.91
Stroop Test Colour (s)	35	235 (165)	185 (87)	36	223 (93)	179 (56)	3 (−27, 33)	0.84
Difference in time (s)	23	110 (99)	97 (64)	27	97 (64)	71 (43)	21 (−4,47)	0.09
Block design (accuracy)	37	4.9 (8.2)	7.6 (8.6)	39	7.3 (7.8)	11.0 (10.0)	−1.5 (−4.5, 1.6)	0.35
Oral fluency (words)	37	3.4 (3.6)	3.0 (2.7)	39	4.1 (5.7)	3.2 (3.0)	−0.1 (−1.4,1.2)	0.91
Oral fluency (categories)	37	18.6 (5.9)	20.0 (5.8)	39	18.1 (6.6)	21.4 (4.9)	−1.6 (−3.7, 0.6)	0.15
Coughlan learning tasks (verbal; items recalled)	37	34.3 (14.2)	38.9 (16.2)	39	30.3 (11.0)	39.6 (14.8)	−3.5 (−9.4, 2.3)	0.23
Coughlan learning task (visual; items recalled)	36	16.8 (9.9)	24.7 (12.0)	37	20.1 (11.5)	24.3 (14.2)	2.5 (−2.9,7.8)	0.36

### Adherence and tolerability

In the study, there were no statistically significant differences in the methotrexate-related side effect checklist between groups. The greatest group difference was nausea occurring in 19.1% of the methotrexate group and 6.7% of the placebo group (Table 4). Dry cough occurred in 10.6% with methotrexate and 4.4% placebo. Oral ulceration and sore throat were more common in the placebo group than those on methotrexate. One participant taking methotrexate developed probable treatment-related symptoms and was later found to have non-critical leukopenia but they had moved and were lost to follow-up. Mean leucocyte count did not change significantly in either group. There were no other adverse reactions or events and no clinically significant abnormalities on renal or hepatic function tests emerged for any of the participants. Eight participants withdrew from each arm but all eight of the placebo group withdrew or were lost to follow up before taking trial medication whereas 5/8 in the methotrexate group dropped out after taking trial treatment, two after 2 weeks (i.e., two doses), one after 4 weeks and two after 8 weeks.

**Table 4:** Side effects.

	Methotrexate *n* = 45		Placebo *n* = 47	
	*N*	%	*N*	%
Rash	0	0.0	1	2.2
Oral ulceration	1	2.1	3	6.7
Nausea*	9	19.1	3	6.7
Vomiting	4	8.5	4	8.9
Diarrhoea	0	0.0	0	0.0
New or increasing dyspnoea	4	8.5	1	2.2
New or worsening dry cough	5	10.6	2	4.4
Severe sore throat	1	2.1	3	6.7
Abnormal bruising	0	0.0	0	0.0
Abdominal pain	4	8.5	3	6.7
Hair loss	2	4.3	2	4.4
Total^a^	33	63.8	22	48.9

^*^p = 0.10 chi square with Yates correction.

^a^*p* = 0.06 chi square.

## Discussion

We report on the first randomised study to assess the feasibility, tolerability and efficacy of methotrexate in patients with schizophrenia. Recruitment was satisfactory from a population of patients in contact with clinical teams and taking antipsychotic drugs. Approximately 70% of those eligible agreed to participate. Our estimated dropout rate of 10% based on our previous studies proved optimistic, nevertheless retention at 82% over 12 weeks compares favourably with antipsychotic drug trials^33^. Furthermore, dropouts were unrelated to reported side effects although poor tolerability may have contributed to the five dropouts from the methotrexate group that occurred after starting trial medication. Nevertheless, the recruitment, retention and tolerability data suggest that a larger study is feasible at the low 10-mg dose. One case of non-critical leukopenia was detected after two doses and this is a recognised occurrence with low-dose methotrexate. Neutropenia in patients receiving low-dose methotrexate ranges from 1.4–7% in trials of inflammatory rheumatic disease^34^. Attrition rates of up to 15% have been reported due to leukopenia, liver function test dyscrasia and/or gastrointestinal side effects and would need to be factored into trial design with equivalent doses^35^.

Methotrexate appeared to exert a selective benefit on positive symptoms in early schizophrenia with no effect on negative symptoms or on cognitive performance but with an overall improvement in general and total symptoms and in general functioning. The study was not primarily designed to detect the efficacy of methotrexate but the effects were statistically significant and robust to examination for confounds such as baseline differences or differential dropouts. The groups were well-matched demographically and in terms of antipsychotic treatment. It seems unlikely that the finding is due to a pharmacokinetic interaction, which increased plasma levels of antipsychotics; methotrexate is excreted largely unchanged and so does not block or induce CYP450 enzymes that metabolise antipsychotic drugs^36^. However, future studies could consider monitoring drug concentrations. There was no effect on the mild–moderate negative symptoms as measured by the PANSS subscale nor on tests of cognition. However, the mostly borderline improvements on social and recreational activity could be an indicator of possible benefit in patients with severe negative symptom impairments especially with evidence of immune dysfunction. There is weak evidence that folic acid (taken by all participants) decreases negative symptoms and this might have obscured a small group difference^37^. A much larger trial would be necessary to have a high probability of repeating our finding on positive symptoms but this would not increase the magnitude of the small clinically unimportant placebo-drug difference. The patients in this study had mild to moderately severe symptoms and studies in more severely ill patients are more likely to reveal definitive efficacy. Furthermore, we used the lowest clinically effective dose in autoimmune disorders, which often needs to be increased and greater doses might also produce greater effects in schizophrenia. However, the health risks of methotrexate are substantial and require careful monitoring, which would rule out large unfocussed trials of greater potentially more effective doses in schizophrenia.

Low-dose methotrexate is a standard treatment in a number of autoimmune disorders that share risk with schizophrenia^18^. The possible efficacy of methotrexate in both disorders, even if very partial in schizophrenia, suggests that they may share aspects of pathogenesis. In autoimmune disorders it is clear that dysfunctional Tregs fail to control immune activation^15^; a similar dysfunction in schizophrenia would account for the systemic low-grade inflammation associated with schizophrenia in studies of peripheral hsCRP and cytokines^38^. Indeed evidence for Treg cell dysfunction is beginning to appear; FoxP1 (critical for Treg function) is a GWAS schizophrenia risk gene^39^ and Treg cells from 40 patients showed a substantial functional impairment as in autoimmune disorders^40^. Treg cells in health occur in the meninges and brain lymphatics and have an important role in immune tolerance and surveillance^41^. For example, activated astroglia secrete chemokines that attract Treg cells into the brain parenchyma and in turn Tregs reduce astrogliosis by secreting an epidermal growth factor^42^. Recent studies using advanced cell-specific expression profiling in human post-mortem brain suggest that astroglial activation is present in schizophrenia whereas microglial gene sets are not overexpressed^43^. We propose that methotrexate may improve psychotic symptoms by promoting Treg-mediated restraint of astroglial inflammation in schizophrenia. Better characterisation of Treg function and cell-mediated immunity in schizophrenia is essential for progress in immunological approaches to therapy. Further studies with methotrexate will require comprehensive immunophenotyping to minimise the number exposed to a drug that can have serious side effects and to identify responsive subtypes. New PET radioligands that recognise the state of astroglial and microglial activation will play an increasingly import role in identifying targets for immunomodulatory drugs^11^.

In summary, we report the first clinical trial of the immunosuppressant drug methotrexate in patients with schizophrenia and the first evidence that a drug with known efficacy in autoimmune disorders with actions on cell-mediated mechanisms, may be effective in reducing psychotic symptoms. Further studies would appear to be feasible and are necessary to identify whether methotrexate has clinically important effects that outweigh the risks, such as in treatment refractory illnesses, severe negative symptoms or in patients with evidence of identifiable immune abnormalities such as impaired Treg function.

## Supplementary information

Supplementary Table ST1 and ST2
