# Correction: A randomised clinical trial of methotrexate points to possible efficacy and adaptive immune dysfunction in psychosis

**DOI:** 10.1038/s41398-021-01449-w

**Published:** 2021-09-01

**Authors:** I. B. Chaudhry, M. O. Husain, A. B. Khoso, M. I. Husain, M. H. Buch, T. Kiran, B. Fu, P. Bassett, I. Qurashi, R. ur Rahman, S. Baig, A. Kazmi, F. Corsi-Zuelli, P. M. Haddad, B. Deakin, N. Husain

**Affiliations:** 1 Faculty of Biology, Medicine and Health, Division of Neuroscience and Experimental Psychology, School of Biological Sciences, University of Manchester, Manchester Academic Health Science Centre, Manchester, M13 9PT UK; 2 Dow University of Health Sciences, Karachi, Pakistan; 3 Ziauddin University Hospital, Karachi, Pakistan; 4 Centre for Addiction and Mental Health, Toronto, ON Canada; 5 Department of Psychiatry, University of Toronto, Toronto, ON Canada; 6 Pakistan Institute of Living and Learning, Karachi, Pakistan; 7 National Institute of Health Research Leeds Biomedical Research Centre, Leeds Teaching Hospitals NHS Trust, Leeds, UK; 8 Institute of Cellular Medicine, Newcastle University and National Institute for Health Research Newcastle Biomedical Research Centre, Newcastle upon Tyne Hospitals NHS Foundation, Newcastle upon Tyne, UK; 9 School of Data Science, Fundan University, Shanghai, China; 10 Stats Consultancy, Amersham, UK; 11 Faculty of Biology, Medicine and Health, Division of Psychology and Mental Health, School of Biological Sciences, University of Manchester, Manchester Academic Health Science Centre, Manchester, M13 9PT UK; 12 Baqai University, Karachi, Pakistan; 13 Karwan e Hayat, Karachi, Pakistan; 14 Division of Psychiatry, Department of Neuroscience and Behaviour, Ribeirão Preto Medical School, University of São Paulo (FMRP—USP), São Paulo, São Paulo, 14048-900 Brazil; 15 Hamad Medical Corporation, Doha, Qatar

**Keywords:** Schizophrenia, Neuroscience

Correction to: *Translational Psychiatry*

10.1038/s41398-020-01095-8 published online 30 November 2020

The original version of this article unfortunately contained an error in the COI section. Unfortunately, P. M. Haddad’s COI statement was missed in the PDF although it was provided by the author. We apologize for this error. P. M. Haddad’s COI statement should be: PMH has received personal fees from Janssen, Lundbeck, NewBridge Pharmaceuticals, Otsuka, and Sunovion outside the submitted work.

