# Supplementary material for: A randomised clinical trial of methotrexate points to possible efficacy and adaptive immune dysfunction in psychosis
Source: Transl Psychiatry. 2020 Nov 30;10:415. doi: 10.1038/s41398-020-01095-8 (PMC7705702; doi:10.1038/s41398-020-01095-8)
Supplement: Supplementary file 1 — Supplementary Table ST1 and ST2 [file 41398_2020_1095_MOESM1_ESM.docx]

|  | Placebo | Methotrexate |
| --- | --- | --- |
| **Second generation antipsychotics** | |  |
| risperidone | 23 | 28 |
| olanzapine | 19 | 17 |
| aripiprazole | 1 | 0 |
| quetiapine | 0 | 2 |
|  |  |  |
| **First generation antipsychotics** | |  |
| fluphenazine depot | 8 | 5 |
| haloperidol | 7 | 4 |
| trifluoperazine | 1 | 1 |
| Chlorpromazine | 1 | 0 |
|  |  |  |
| Two antipsychotics | 13 | 12 |
|  |  |  |
| **Other** |  |  |
| antidepressant | 8 | 6 |
| benzodiazepine | 19 | 17 |
| procyclidine | 35 | 29 |
| valproate | 17 | 15 |
| lithium | 3 | 2 |

**Table ST1 Antipsychotic drug treatment**

**Table ST2 Social Function Scale; total subdomain scores**

| SFS subscales, specimen items |  | Placebo (n=39) | |  | Methotrexate (n=37) | |  | Anova |
| --- | --- | --- | --- | --- | --- | --- | --- | --- |
|  |  | Mean | SEM |  | Mean | SEM |  | p |
| Social engagement, time alone, conversations | Baseline | 11.2 | 0.5 |  | 10.5 | 0.5 |  |  |
|  | 3 month | 12.2 | 0.5 |  | 12.1 | 0.5 |  | 0.92 |
|  |  |  |  |  |  |  |  |  |
| Interpersonal behaviour, quality, number of friends | Baseline | 13.7 | 0.9 |  | 12.8 | 1.0 |  |  |
|  | 3 month | 15.1 | 1.0 |  | 17.0 | 1.0 |  | 0.08 |
|  |  |  |  |  |  |  |  |  |
| Pro-social activities, number with others e.g. sport, outings | Baseline | 8.7 | 1.0 |  | 8.0 | 1.0 |  |  |
|  | 3 month | 9.9 | 1.2 |  | 12.6 | 1.2 |  | 0.03 |
|  |  |  |  |  |  |  |  |  |
| Recreational activities, hobbies, interests, pastimes | Baseline | 6.7 | 0.7 |  | 6.6 | 0.7 |  |  |
|  | 3 month | 7.8 | 0.7 |  | 8.5 | 0.7 |  | 0.06 |
|  |  |  |  |  |  |  |  |  |
| Independence (Competence)  Skills for independent living | Baseline | 27.5 | 1.4 |  | 26.2 | 1.4 |  |  |
|  | 3 month | 28.9 | 1.3 |  | 31.2 | 1.4 |  | 0.11 |
|  |  |  |  |  |  |  |  |  |
| Independence (Performance)  Skills for independent living | Baseline | 17.8 | 1.4 |  | 18.4 | 1.4 |  |  |
|  | 3 month | 21.8 | 1.3 |  | 22.0 | 1.4 |  | 0.93 |

Anova = 3 month score by treatment group, covarying baseline. p = main effect of treatment
